# Assessing spatial learning and memory in mice: Classic radial maze versus a new animal-friendly automated radial maze allowing free access and not requiring food deprivation

**DOI:** 10.3389/fnbeh.2022.1013624

**Published:** 2022-09-30

**Authors:** Joel Kohler, Jie Mei, Stefanie Banneke, York Winter, Matthias Endres, Julius Valentin Emmrich

**Affiliations:** ^1^Department of Neurology and Experimental Neurology, Neurocure Cluster of Excellence, Charité – Universitätsmedizin Berlin, Berlin, Germany; ^2^The Brain and Mind Institute, University of Western Ontario, London, ON, Canada; ^3^Department of Computer Science, University of Western Ontario, London, ON, Canada; ^4^German Federal Institute for Risk Assessment (BfR), German Center for the Protection of Laboratory Animals (Bf3R), Berlin, Germany; ^5^Institute of Biology, Humboldt University, Berlin, Germany; ^6^Center for Stroke Research, Charité – Universitätsmedizin Berlin, Berlin, Germany; ^7^Berlin-Brandenburg School for Regenerative Therapies (BSRT), Berlin, Germany; ^8^Berlin Institute of Health (BIH), Berlin, Germany; ^9^German Center for Neurodegenerative Diseases (DZNE), Berlin, Germany; ^10^German Center for Cardiovascular Research (DZHK), Berlin, Germany; ^11^Medical Faculty and University Hospital, Heidelberg Institute of Global Health, University of Heidelberg, Heidelberg, Germany

**Keywords:** memory, spatial learning, behavioral test, radial arm maze (RAM), maze, automation, LPS (lipopolysaccharide)

## Abstract

The radial arm maze (RAM) is a common behavioral test to quantify spatial learning and memory in rodents. Prior attempts to refine the standard experimental setup have been insufficient. Previously, we demonstrated the feasibility of a fully automated, voluntary, and stress-free eight-arm RAM not requiring food or water deprivation. Here, we compared this newly developed refined RAM to a classic manual experimental setup using 24 female 10–12 weeks old C57BL/6J mice. We used a lipopolysaccharide (LPS)-induced model of systemic inflammation to examine long-term cognitive impairment for up to 13 weeks following LPS injection. Both mazes demonstrated robust spatial learning performance during the working memory paradigm. The refined RAM detected spatial learning and memory deficits among LPS-treated mice in the working memory paradigm, whereas the classic RAM detected spatial learning and memory deficits only in the combined working/reference memory paradigm. In addition, the refined RAM allowed for quantification of an animal’s overall exploratory behavior and day/night activity pattern. While our study highlights important aspects of refinement of the new setup, our comparison of methods suggests that both RAMs have their respective merits depending on experimental requirements.

## Introduction

Replacement, Reduction, and Refinement constitute the 3R principles which have guided animal behavior research since its introduction in 1959 (Russell and Burch, 1959). Although considerable progress has been made in the past decades toward achieving these principles (Fenwick et al., 2009; Bayne et al., 2015; Lewis, 2019; Lee et al., 2020), reducing an animal’s pain, suffering, and distress while ensuring scientific validity of results remains a constant challenge.

Mazes are commonly used for behavioral tests to assess spatial learning and memory in rodents. Among a variety of different types, the eight-arm radial arm maze (RAM) is one of the most frequently used methods. It was introduced by Olton and Samuelson (1976) and has since been used to test the cognitive performance of mice in various disease models including Alzheimer’s disease (Choi et al., 2018), posttraumatic stress disorder (El Hage et al., 2006), depression (Yadav et al., 2013), and sepsis (Semmler et al., 2007; Weberpals et al., 2009; Anderson et al., 2015).

Traditionally, testing in the classic RAM is performed manually and requires food and/or water deprivation. For animals being tested in the classic RAM, manual handling and food and/or water deprivation may result in a substantial degree of stress. The classic RAM setup thereby also introduces a variety of possible confounders. The close olfactory, visual, auditory, and tactile interactions between experimenter and animal may result in anxiety and handling stress among experimental animals, which, in turn, may endanger the reproducibility of an experiment (Hurst and West, 2010; Gouveia and Hurst, 2017; Gulinello et al., 2019). Other confounding effects are caused by water and/or food deprivation which are commonly used to motivate foraging (Vorhees and Williams, 2014). In addition, testing animals in the RAM can be quite time-consuming for the experimenter as animals cannot be tested simultaneously.

To address these shortcomings of the classic RAM, we recently demonstrated the feasibility of a fully automated, voluntary, and handling-free refined version of the RAM allowing free access and not requiring food or water deprivation (Mei et al., 2020). There have been various other attempts to refine the classic RAM setup including automated detection of an animal’s location or pellet intake using cameras, photoelectric or pressure sensors as well as automation of some mechanical parts of the RAM but none were handling-free, allowed free access and did not require food or water deprivation (Peele and Baron, 1988; Miyakawa et al., 2001; Dubreuil et al., 2003; Brillaud et al., 2005; Risher et al., 2013).

With this study, we aimed to compare the classic manual setup with the refined automated version of the RAM. We used an established mouse model of lipopolysaccharide (LPS)-induced systemic inflammation (Barichello et al., 2019; Savi et al., 2021), which has been shown to elicit long-term memory impairment in rodents in a classic RAM setup (Semmler et al., 2007; Weberpals et al., 2009), to induce long-term cognitive deficits. By reflecting on the respective merits of the two methods, we contribute to the refinement of future RAM experiments supporting the application of the third of the three 3R principles.

## Materials and methods

### Ethical statement

All experimental procedures were reviewed and approved by the State Office for Health and Social Affairs [Landesamt für Gesundheit und Soziales (LaGeSo), Berlin, Germany], Berlin (G290/15) and were carried out in accordance with the German animal protection law and local welfare guidelines at the German Federal Institute for Risk Assessment [Bundesinstitut fu̇r Risikobewertung (BfR), Berlin, Germany]. Reporting of the study complies with the ARRIVE 2.0 guideline (Percie du Sert et al., 2020).

### Animals, housing, husbandry, and setting

We used female C57BL/6J mice, that were 10–12 weeks old at the beginning of the study, obtained from Charles River Laboratories, Sulzfeld, Germany at the age of 6–8 weeks. Animals were kept under specific-pathogen-free conditions according to FELASA recommendations. Housing conditions were as follows: Room temperature 23 ± 1°C, humidity 60 ± 5%, inverse 12:12 h light:dark cycle [lights on: 20:00, lights off: 8:00)]. Animals were group-housed in type III polycarbonate cages (1290D Euro standard Type III, Techniplast, Italy) equipped with environmental enrichment tools (red transparent plastic nest box and nesting material), with *ad libitum* access to food (autoclaved pellets; Lasvendi, LASQCdiets TM ROD16-H) and water. All persons entering the laboratory rooms wore single-use coveralls (Microgard 1,500, Ansell Microgard, Kingston Upon Hull, UK), gloves and surgical masks to reduce potential olfactory confounding effects. We cleaned the arms of each maze daily to remove droppings. Upon completion of each experimental group, we cleaned and disinfected the RAMs thoroughly using warm water, soap, and an alcohol-based disinfectant. All procedures and experiments were performed in the same facility as where animals were housed.

### Apparatus

**FIGURE 1:**
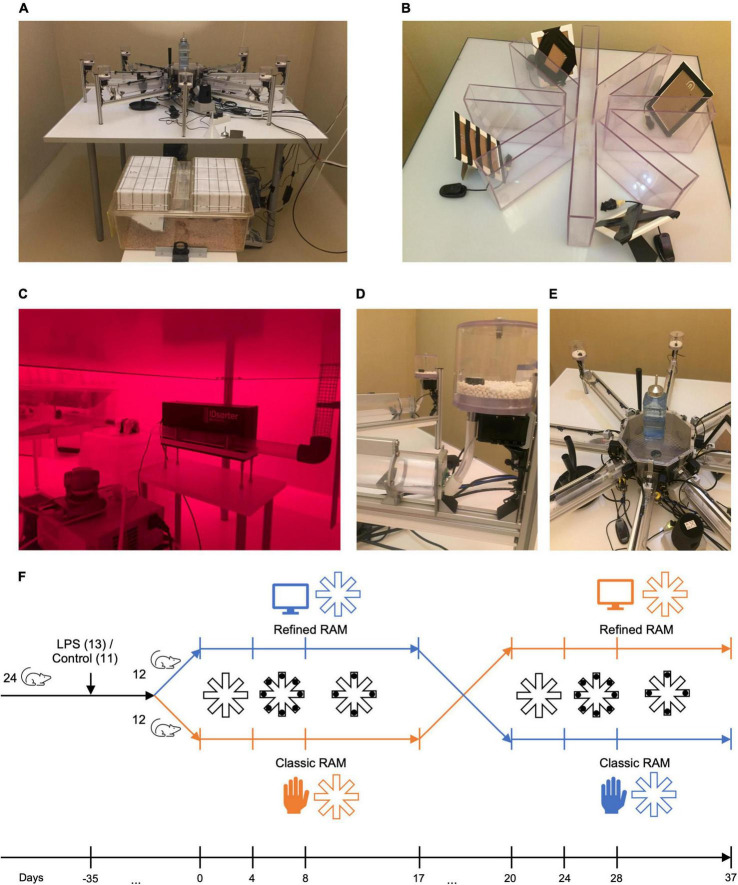
Setup of the refined and classic radial arm mazes (RAM) and timeline. **(A)** Refined RAM connected to the animals’ home cage; **(B)** Setup of the classic RAM including extra-maze visual cues; **(C)** Radio-frequency identification-based animal sorter device and climbing wire connecting the home cage to the refined RAM (pictured under red light conditions during the animal’s active phase); **(D)** Automated sucrose enriched pellet dispenser; **(E)** Central platform of the refined RAM including extra-maze visual cues; **(E)** Setup of the classic RAM showing extra-maze visual cues; **(F)** Timeline of the experiments in the refined and the classic RAM. We used a cross-over design; one group of animals was tested in the refined RAM first and continued in the classic RAM after a washout phase of 3 days and *vice versa*. Experimental phases included habituation (refined RAM: habituation cage, classic RAM: maze, with pellets distributed in the home cage/maze), working memory paradigm (eight arms baited), and combined working/reference memory paradigm (four arms baited). RAM, radial arm maze.

The classic RAM was made from polycarbonate and consisted of eight equally spaced, rectangular arms (length: 30 cm, width: 5 cm, and height: 20 cm) that were open at the top and which extended from a central octagonal platform (13 cm across) (Figure 1B). At the end of each arm, there was a small cavity in which a pellet was placed. The experimenter manually placed the animal onto the central platform of the maze using a containment box to minimize handling stress. Likewise, upon completion of an experimental session, animals were removed from the classic maze using a containment box. We scheduled experiments in the classic RAM at the same time each day during the active (lights off) phase.

We described the refined RAM previously (Mei et al., 2020). In short, it consisted of eight transparent tubes radiating outwards from an octagonal holding platform (Figures 1A,E). An automated pellet dispenser was located at the end of each arm (Figure 1D) which dispensed a pellet once an animal entered a correct arm. The maze was connected to a standard home cage *via* an animal sorter device (Figure 1C). Animals could freely access the refined RAM from their home cage at any time of the day. Only one home cage (containing six animals with a random combination of LPS-treated and control animals) was connected to the refined RAM at any given time. An RFID reader and photoelectric sensors allowed to determine the location of an animal within the refined RAM. The animal sorter device ensured that only one animal could enter the maze at a given time.

For both mazes, illuminated visual cues including different objects like a candleholder and picture frames presenting various geometrical patterns were placed next to the maze as distal cues. The central platforms contained tactile cues constituting local cues for additional tactile orientation. We used sucrose enriched pellets (Purified Rodent Tablets 5TUL) from Test Diet, Richmond, USA.

### Transponder implantation

To allow for animal identification, radio-frequency identification (RFID) transponders were implanted subcutaneously in all mice at least 2 weeks prior to the beginning of the experiments. Glass-covered, biocompatible RFID transponders (dimensions: 2.1 mm × 12 mm; model: passive 125 kHz glass transponder; EURO I.D. Identifikationssysteme GmbH & Co., KG, Frechen, Germany) with individual identification numbers were sterilized and loaded in an applicator device. We implanted the transponders subcutaneously in the nuchal region. Anesthesia was induced with 3% isoflurane delivered in 100% oxygen for 45 s before the implantation procedure. For analgesia animals received meloxicam (1 mg/kg; Sigma-Aldrich, St. Louis, MO, USA) subcutaneously once during the procedure. Following implantation, mice were observed for up to 48 h for signs of complications. Presence and functionality of the RFID transponder was checked before the start of the experiment.

### Experimental design

This was a randomized, blinded method-comparison study. We conducted an *a priori* sample size calculation based on findings from previous classic RAM studies assessing long-term cognitive deficits following LPS-injection using G*Power (Faul et al., 2007). The study was designed with 80% power to detect a relative 25% difference in combined working/reference memory performance. *A priori* power analysis using a repeated measures ANOVA with Tukey’s *post-hoc* test under the following assumptions α = 0.05, β = 0.2 and based on mean and SD obtained from preliminary experiments determined the number of required experimental units at 12 animals per group.

First, animals were randomly assigned to one of two treatment groups (LPS-treated or control group). The number of animals per group after randomization was 13 in the LPS-treated group and 11 in the control group. Second, animals were randomly assigned to one of two experimental groups because we used a cross-over design (for an illustration of the experimental design, see Figure 1F). The first experimental group began in the refined RAM and was subsequently tested in the classic RAM. The second experimental group began in the classic RAM and was subsequently tested in the refined RAM. We performed this cross-over testing twice with two groups of 12 animals in sequence: The first group of 12 animals was randomly divided in two subgroups of six animals. After both subgroups had finished the experiments in both mazes, the second group of 12 animals followed in the same manner. The six animals of one subgroup remained together in one cage for the entire time of the experiment without contact to animals from other cages. Due to technical reasons, the duration between injection and start of experiments varied from 5 to 10 weeks for the working memory paradigm and 7–13 weeks for the combined working/reference memory paradigm for both mazes.

### Methods to prevent bias

Animals were randomized to treatment groups, experimental groups, and to rewarding arm pattern in the combined working/reference memory paradigm using the Research Randomizer tool.^1^ The researcher conducting the experiments was blinded regarding treatment group assignment until the end of data analysis.

### Experimental procedures

#### Treatments

To induce a systemic inflammatory response, animals were treated with LPS, a cell wall component of Gram-negative bacteria. LPS (from *Salmonella enterica* serotype, Lot # 056M4115V, Sigma-Aldrich St. Louis, MO, USA) at a dose of 1.5 mg/kg or physiological phosphate-buffered saline solution were administered intraperitoneally on two consecutive days at the beginning of the active (i.e., light-off) phase at 8:00 with a volume of 10 μl/g. After injection, animals were monitored closely using a sickness score adapted from the murine sepsis score. These procedures were performed as previously described (Mei et al., 2018, 2019).

#### Food deprivation

For the classic RAM, animals were food deprived for 8 h before testing. Animals were weighed twice daily. First, before the food was removed and second, before the beginning of the testing session. If weight loss exceeded 15% compared to baseline weight, food deprivation would have been stopped until baseline weight had been regained. Recorded weight loss never exceeded 5%. We did not food deprive animals for the refined RAM.

#### Habituation phase

We habituated animals to the experimental setup for 3–5 days. For the classic RAM, we placed sucrose enriched pellets all over the maze and animals were allowed to move freely within the maze for up to 30 min per day. For the refined RAM, we used a habituation cage followed by exploration of the refined RAM as described previously (Mei et al., 2020).

#### Working memory paradigm

The actual experiment consisted of a working memory paradigm and a combined working/reference memory paradigm. During the working memory paradigm, a sucrose enriched pellet was placed by the end of each of the eight arms of the maze (manually in the classic RAM; the refined RAM dispensed a pellet when an animal visited a correct arm for the first time during a session). The working memory paradigm lasted for 4 days. We assessed spatial working memory performance during the working memory paradigm by considering an animal’s reentry into a previously visited arm as a working memory error. We present correct choices (i.e., first entries to reward-baited arms during one experimental session) as three different ratios (see the section “Behavioral parameters”).

#### Combined working/reference memory paradigm

During the combined working/reference memory paradigm, a sucrose enriched pellet was placed by the end of each of the four randomly selected arms of the maze. The configuration of the four randomly selected arms remained the same for every individual mouse throughout the combined working/reference memory paradigm. The combined working/reference memory paradigm lasted for 9 days and began immediately after the end of the working memory paradigm in the refined RAM and on the day following the end of the working memory paradigm in the classic RAM. We considered an animal’s reentry into a previously visited baited arm as spatial working memory error. In addition, we considered a (re-)entry into an unbaited arm as spatial reference memory error.

#### Sessions

In the classic RAM, animals were tested once per day during working memory paradigm and combined working/reference memory paradigm in the classic RAM. Animals could voluntarily enter the refined RAM for up to ten times per day during both phases. Start and end of sessions were defined as follows. For the classic RAM, a session started when the mouse was released in the central platform of the maze, while for the refined RAM a session started when an animal voluntarily entered the maze. Sessions were terminated upon task completion (visiting all eight arms in the working memory paradigm and visiting all four baited arms in the combined working/reference memory paradigm) or when 10 min elapsed. At the end of a session (when task was completed or when the time-out limit was reached), in the classic RAM mice were retrieved by the experimenter and returned to their home-cage, while in the refined RAM all arms closed apart from the one containing the mouse. When the mouse left the last arm and returned to the central platform, the last visited arm closed, too, leaving available only the path leading back to the home-cage.

### Behavioral parameters

Our outcomes were working memory errors and reference memory errors. In addition, we report the time animals needed to complete one session (i.e., entering all baited arms) and three different ratios: the ratio of correct entries to the sum of all entries [calculation: correct entries to reward-baited arms divided by the sum of all arm entries; i.e., correct entries ratio (all arms visited)], the ratio of correct entries within the first four arm visits [calculation: correct entries to reward-baited arms within the first four arms visits of a session divided by the number of reward-baited arms (eight in the working memory paradigm, four in the combined working/reference memory paradigm), i.e., correct entries ratio (first four arms visited)], and the ratio of correct entries within the first eight arm visits [calculation: correct entries to reward-baited arms within the first eight arms visits of a session divided by the number of reward-baited arms, i.e., correct entries ratio (first eight arms visited)] (Wenk, 2004). This approach, normalizing the number of correct entries by the total number of baited arms, avoided underscoring the performance of mice in the eight arm ratio in the relative comparison with the four arm ratio. This approach guarantees that each addition of a correct entry is correctly scored as an increase in the ratio. In the refined RAM, since multiple daily sessions were performed by mice, the values of the behavioral parameters were averaged across daily sessions in order to obtain a single daily value. To ensure spatial learning, we excluded the data generated by mice which had two or fewer maze entries during working memory paradigm or three or fewer maze entries during combined working/reference memory paradigms, respectively.

### Data analysis and statistical methods

All data values are shown in mean ± standard deviation (SD) unless indicated otherwise. All experiments in the classic RAM were video recorded and analyzed manually. A custom-made software controlled the refined RAM and recorded experimental data.

Statistical analysis was performed using SPSS (Version 26.0). We analyzed data using linear mixed models. We used random intercept models that account for the clustering of measures within individuals. The measures of the behavioral outcomes served as dependent variable; treatment (LPS/control), maze type (refined/classic RAM), experimental order of maze type (first refined, then classic, or first classic, then refined) and interactions of treatment*time, maze type*time, treatment*maze type, and maze type*experimental order of maze type as factors; and time (days) as covariate. Deviation from normal distribution was checked with histograms and we log-transformed the data before analysis if they were not sufficiently normally distributed. We report model-based marginal means and group differences with 95% confidence intervals (CIs) as well as within-group differences. A two-sided significance level of α = 0.05 was used.

## Results

Thirteen animals were randomized to the LPS-treatment group; eleven animals were randomized to the control group. One animal had to be killed following LPS-injection because it exceeded the pre-defined sickness severity cut-off score.

### Refined radial arm maze

**FIGURE 2:**
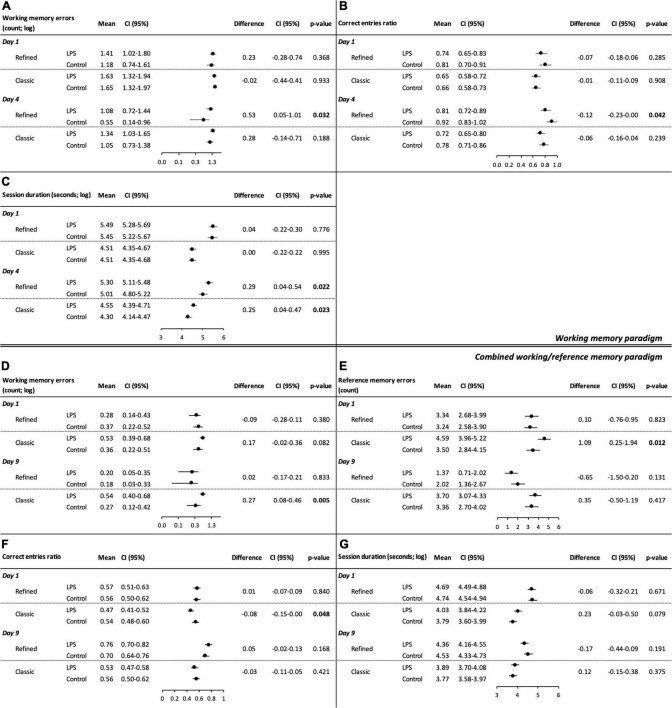
Cognitive performance in the refined and classic radial arm mazes during the working memory paradigm **(A–C)** and the combined working/reference memory paradigm **(D–G)**. Separate linear mixed model analyses were conducted. Model-derived estimated marginal means and group differences for **(A)** working memory errors (log-transformed), **(B)** correct entries ratio (all arms visited), and **(C)** session duration (log-transformed) on the first (1 day) and last day (4 day) of the working memory paradigm are shown. Model-derived estimated marginal means and group differences for the combined working/reference memory paradigm on the first (1 day) and last day (9 day) of the paradigm: **(D)** working memory errors (log-transformed), **(E)** reference memory errors, **(F)** correct entries ratio (all arms visited), and **(G)** session duration (log-transformed).

**FIGURE 3:**
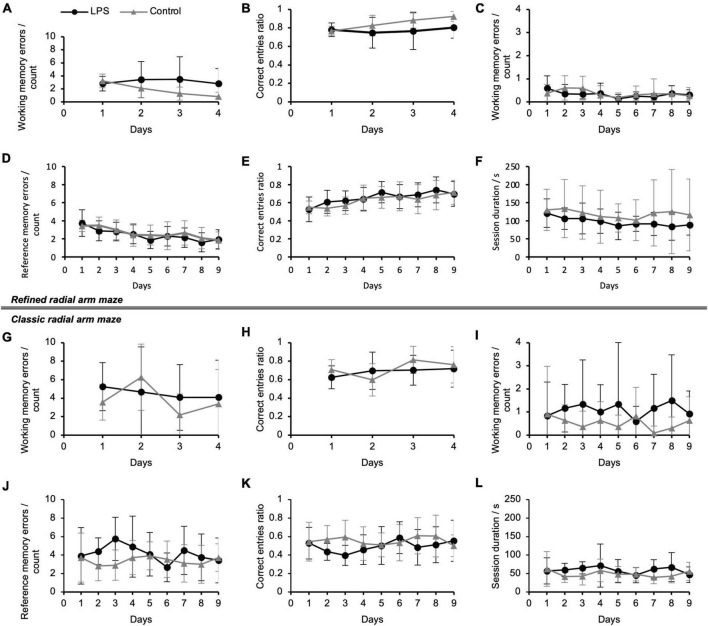
Spatial working and reference memory performance of mice following lipopolysaccharide (LPS)-injection in the refined radial arm maze (RAM) during working memory paradigm **(A,B)** and combined working/reference memory paradigm **(C–F)** and in the classic RAM during working memory paradigm **(G,H)** and combined working/reference memory paradigm **(I–L)**. Refined RAM: **(A)** Average number of working memory errors per animal per day (re-entries into an arm which had already been visited during a session) during the working memory paradigm; **(B)** Correct entries ratio (all arms visited) during the working memory paradigm; the ratio expresses the fact that the animals reached the maximum number of correct entries in most of the sessions (also in panel **E**); **(C)** Average number of working memory errors per session during the combined working/reference memory paradigm; **(D)** Average number of spatial reference memory errors (i.e., number of entries and re-entries to unbaited arms per session) per animal per day; **(E)** Correct entries ratio (all arms visited) during the combined working/reference memory paradigm; **(F)** Average session duration from entering the refined RAM until all baited arms had been visited and the animal exited the RAM per animal per day; maximum session duration was: 10 min. Working memory paradigm: *N* = 9 (LPS-treated group), *N* = 6 (control group); combined working/reference memory paradigm: *N* = 11 (LPS-treated group), *N* = 11 (control group). Classic RAM: **(G)** Working memory errors and **(H)** correct entries ratio (all arms visited) during the working memory paradigm; **(I)** working memory errors, **(J)** reference memory errors, **(K)** correct entries ratio (all arms visited) and **(L)** session duration during the combined working/reference memory paradigm. *N* = 12 (LPS-treated group), *N* = 11 (control group). Data are presented as mean (±SD). Half of the individuals tested with the refined RAM had previously been trained on the classic RAM and vice versa. RAM, radial arm maze.

Figures 2A–C and Supplementary Figures 2A,B show model-derived adjusted means for each treatment group as well as adjusted treatment effects (group differences) on the first and last days of the working memory paradigm (eight arms baited). The corresponding descriptive statistics are displayed in Figures 3A–F and Supplementary Figures 3A–D. Spatial learning performance is summarized in Supplementary Table 1.

During the working memory paradigm, we observed treatment group differences on the last day of the paradigm for working memory errors [treatment effect: 0.53 (log-transformed), 95% CI: 0.05–1.01, *P* = 0.032; Figure 2A], correct entries ratio (all arms visited) (treatment effect: −0.12, 95% CI: −0.23–0.00, *P* = 0.042; Figure 2B), and session duration [treatment effect: 0.29 (log-transformed), 95% CI: 0.04–0.54, *P* = 0.022; Figure 2C], which indicates spatial learning and memory deficits among LPS-treated animals. There was no relevant treatment group difference on the first day of the paradigm neither for session duration, working memory errors nor for correct entries ratios. Working memory errors and session duration decreased over time among control animals [working memory errors: difference day 1 day 4: 0.63 (log-transformed), 95% CI: 0.06–1.19, *P* = 0.030; session duration: difference day 1 day 4: 0.44 (log-transformed), 95% CI: 0.14–0.74, *P* = 0.004; Supplementary Table 1], indicating spatial learning performance. There was no effect of time on working memory errors among LPS-treated animals.

Figures 2D–G and Supplementary Figures 2C,D show model-derived adjusted means for each treatment group as well as adjusted treatment effects (group differences) on the first and the last days of the combined working/reference memory paradigm (four arms baited). During the combined working/reference memory paradigm, we found no relevant group differences. Reference memory errors (LPS-treated animals: difference day 1 day 9: 1.97, 95% CI: 1.09–2.85, *P* < 0.001; control animals: difference day 1 day 9: 1.22, 95% CI: 0.34–2.10, *P* = 0.007), correct entries ratio (first four arms visited) (LPS-treated animals: difference day 1 day 9: −0.24; 95% CI: −0.34–0.14, *P* < 0.001; control animals: difference day 1 day 9: −0.14, 95% CI: −0.24–0.04, *P* = 0.006), correct entries ratio (all arms visited) (LPS-treated animals: difference day 1 day 9: −0.19; 95% CI: −0.27–0.11, *P* < 0.001; control animals: difference day 1 day 9: −0.14, 95% CI: −0.22–0.07, *P* < 0.001), and session duration (LPS-treated animals: difference day 1 day 9: 0.33 (log-transformed), 95% CI: 0.13–0.53, *P* = 0.001; control animals: difference day 1 day 9: 0.21 (log-transformed), 95% CI: 0.01–0.41, *P* = 0.040) improved over time in both treatment groups; correct entries ratio (first eight arms visited) improved among LPS-treated animals (difference day 1 day 9: −0.07; 95% CI: −0.13–0.01, *P* = 0.019) (Supplementary Table 1).

Taken together, these results indicate a subtle deficit in spatial learning and memory among LPS-treated mice compared to control animals. To account for a potential washout of the treatment effect due to multiple daily sessions, we analyzed the first four sessions of the working memory paradigm and the first nine sessions of the combined working/reference memory paradigm, separately. We did not observe a significant difference between treatment groups during the first sessions of a paradigm in the refined RAM (Supplementary Figure 1).

### Classic radial arm maze

During the working memory paradigm, there was a significant treatment group difference for session duration on the last day of the paradigm (treatment effect: 0.25 (log-transformed), 95% CI: 0.04–0.47, *P* = 0.023; Figure 2C). We did not find group differences neither for working memory errors nor for the correct entries ratio (all arms visited) (corresponding descriptive statistics are summarized in Figures 3G–L and Supplementary Figure 3H). Working memory errors [difference day 1 day 4: 0.59 (log-transformed), 95% CI: 0.14–1.04, *P* = 0.010] decreased and correct entries ratio (first eight arms visited) (difference day 1 day 4: −0.10, 95% CI: −0.19–0.01, *P* = 0.026) and correct entries ratio (all arms visited) increased (difference day 1 day 4: −0.13, 95% CI: −0.23–0.02, *P* = 0.021) among control animals (Supplementary Table 1). There was no effect of time on working memory errors among LPS-treated animals.

During the combined working/reference memory paradigm, we observed a treatment effect for working memory errors on the last day of the paradigm [treatment effect: 0.27 (log-transformed), 95% CI: 0.08–0.46, *P* = 0.005; Figure 2D] and for reference memory errors (treatment effect: 1.09, 95% CI: 0.25–1.94, *P* = 0.012; Figure 2E), correct entries ratio (first four arms visited) (treatment effect: −0.09, 95% CI: −0.18–0.00, *P* = 0.047, Supplementary Figure 2C), correct entries ratio (first eight arms visited) (treatment effect: −0.06, 95% CI: −0.12–0.00, *P* = 0.040, Supplementary Figure 2D), and correct entries ratio (all arms visited) (treatment effect: −0.08, 95% CI: −0.15–0.00, *P* = 0.048; Figure 2F), on the first day of the paradigm, respectively. These results indicate spatial learning and memory deficits among LPS-treated animals. There was a trend toward poorer cognitive performance among LPS-treated animals for working memory errors and session duration on the first day of the paradigm, albeit not statistically significant [working memory treatment effect: 0.17 (log-transformed), 95% CI: −0.02–0.36, *P* = 0.082, Figure 2D; session duration treatment effect: 0.23 (log-transformed), 95% CI: −0.03–0.50, *P* = 0.079, Figure 2G]. Apart from a decrease of reference memory errors (difference day 1 day 9: 0.89, 95% CI: 0.03–1.75, *P* = 0.042) and an increase of the correct entries ratio among LPS-treated animals (first four arms visited) (difference day 1 day 9: −0.10, 95% CI: −0.19–0.00, *P* = 0.048), neither LPS-treated nor control animals showed a significant change in performance over time, indicating overall poor spatial learning performance (Supplementary Table 1).

Taken together, these results indicate that LPS-treated animals had subtle cognitive deficits which could be detected both in the refined and classic RAM.

### Activity in the refined radial arm maze

**FIGURE 4:**
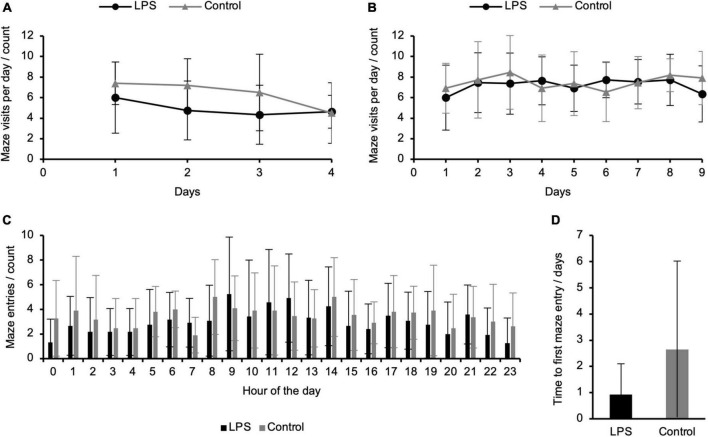
Exploratory activity and day/night activity of control and lipopolysaccharide (LPS)-treated animals in the refined radial arm maze (RAM). **(A)** Average number of individual entries to the RAM per day during the working memory paradigm and **(B)** combined working/reference memory paradigm remained largely unchanged; **(C)** Average sessions per hour of LPS-treated and control animals across all days of the combined working/reference memory paradigm (active phase, lights off: 8.00–20.00; inactive phase, lights on: 20.00–8.00) showed a physiological increase of locomotor activity during the active phase; **(D)** Average latency to first entry to the RAM during the working memory paradigm did not reveal a significant difference between the LPS-treated and the control group. Data are presented as mean (±SD). Working memory paradigm: *N* = 9 (LPS-treated group), *N* = 6 (control group); combined working/reference memory paradigm: *N* = 11 (LPS-treated group), *N* = 11 (control group).

In addition to cognitive performance, continuous data acquisition in the refined RAM allowed us to quantify an animal’s day/night activity pattern and exploratory behavior. During the animals’ active phase (i.e., lights off), both groups entered the maze more frequently than during the inactive phase (i.e., lights on), representing a physiological day/night activity pattern (Refinetti, 2004; Arakawa et al., 2007; Pioli et al., 2014; Saré et al., 2021; Figure 4C). Latency to first entry to the maze as an indicator of exploratory behavior was 2.65 (±3.38) days for the control and 0.92 (±1.18) days for the LPS-treated groups, respectively, indicating within- and between-groups variations whereas the between-groups difference was not significant (Figure 4D). The average number of maze entries per day remained largely unchanged during the duration of the experiment for both groups (Figures 4A,B).

### Data exclusion

Due to a low number of daily maze entries to the refined RAM, we excluded eight animals (three from LPS-treated group; five from control group) during the working memory paradigm and one animal from the LPS-treated group during the combined working/reference memory paradigm. We excluded two of 322 maze visits (0.6%; 1 day for one animal from the LPS-treated groups; 1 day for one animal from control group) to the classic maze due to corrupted video files. We excluded 30 of 402 maze visits to the refined RAM (7.4%; 11/402 = 2.7% from LPS-treated group; 19/402 = 4.7% from control group) during the working memory paradigm and 121 of 1,881 maze visits (6.4%; 66/1,881 = 3.5% from LPS-treated group; 55/1,881 = 2.9% from control group) during the combined working/reference memory paradigm due to errors of the control software of the refined RAM.

## Discussion

The aim of our study was to compare the advantages and limitations of a classic manual eight-arm radial maze with those of a fully automated refined equivalent. A particular strength of our study was that the same animals were used in both apparatuses, which allowed a direct, intra-individual comparison.

During the working memory paradigm, LPS-treated animals demonstrated a worse cognitive performance in the refined RAM but not in the classic RAM. During the combined working/reference memory paradigm, LPS-treated animals performed worse in the classic RAM but not in the refined RAM. Overall, LPS-induced cognitive deficits were subtle. In addition to cognitive performance, which both mazes readily detected, continuous data acquisition in the refined RAM allowed quantification of an animal’s exploratory behavior and day/night activity pattern.

Previous studies in mice found mixed effects of LPS-induced systemic inflammation on cognitive performance in the RAM. In one study, working and reference memory deficits persisted for up to 2 months following 5 mg/kg LPS injection (Weberpals et al., 2009), whereas another study found no effect of 5 mg/kg LPS injection on working memory performance one month after injection (Anderson et al., 2015). In the present study, we used a relatively low LPS dosage (2 × 1.5 mg/kg), which may explain the subtle treatment effect. In addition, the interval between the injections and the beginning of the working memory paradigm was comparatively long (up to 13 weeks) for some animals due to unexpected technical challenges of the refined RAM. A washout of the treatment effect during multiple daily sessions might have further reduced discrimination power in the refined RAM. However, we did not observe a significant difference between treatment groups even during the first sessions of a paradigm in the refined RAM. Future studies should consider limiting the number of daily sessions or the time period during which animals can access the refined RAM to avoid potential washout effects. Using different experimental setups such as object recognition and open field test, others have shown that cognitive function following sepsis improves over time, which supports our findings (Tuon et al., 2008; Comim et al., 2011).

Latency to first entry to the refined RAM appeared to be shorter among LPS-injected animals, albeit not significantly. Previous studies showed decreased exploratory activity following systemic inflammation in rodents (Haba et al., 2012; Anderson et al., 2015; Ye et al., 2019). However, these studies measured exploratory behavior right after LPS-induced systemic inflammation, i.e., during acute sickness behavior. Future studies should assess the potential long-term effects of LPS on exploratory behavior once animals have recovered from acute sickness.

Apart from sensitivity and variations of measurement, other characteristics must be considered for a comprehensive method comparison. In terms of animal welfare, the refined RAM had the important advantage of not requiring food or water deprivation; such deprivation is standard procedure to increase an animal’s reward seeking behavior in the classic RAM. Whereas there have been successful previous attempts to refrain from food deprivation in six-arm and eight-arm radial mazes, none of these mazes allowed free access (Fitzgerald et al., 1988; Haga, 1995; Opitz et al., 1997). In addition, experiments in the refined RAM lasted for up to 3 weeks without manual handling by an experimenter whereas animals required daily handling in the classic RAM. In addition to handling itself, other potentially confounding factors including an experimenter’s level of experience or sex could not affect animal performance in the refined RAM. By lowering an animal’s stress level during cognitive testing, the refined RAM may thus reduce between- and within-subject variations, which, in turn, may improve characterization of cognitive performance and reproducibility of experimental studies. Further studies are needed to quantify the effect of the refined RAM on stress levels.

Another conspicuous difference between the classic and the refined experimental procedure was the time required to set up and run the experiments. While animals in the classic maze required daily handling by an experienced experimenter lasting up to 15 min per animal per day, the refined maze only required a 5 min, basic daily inspection by an animal technician. Thus, while the experimenter spent drastically less time on conducting experiments in the refined RAM, the overall duration of the experiment was around 8 days longer in the refined compared to the classic RAM. This was because animals could voluntarily enter the refined RAM at a time of their choosing, which necessitated to prolong experiments until animals were sufficiently trained. Experimental time could possibly be reduced if experimental pellet feeders provided whole diet pellets, and overall food availability would largely be through these pellet feeders as done in another study using the same automation technology (Caglayan et al., 2021). Regarding the smell of the pellets, the refined RAM holds the advantage of masking the smell when an animal is on the central platform since a pellet is only dispensed at the moment a mouse enters a baited arm. Additionally, in the refined RAM, only one cage at a time is connected to the maze. This should be taken into account when planning experimental designs in which the age of the animals and/or the timing from the treatment is of importance.

The refined RAM as a custom-made device is expensive to purchase and maintain. In the future, however, commercialization of the refined RAM could reduce costs. Finally, the necessity to exclude data was comparatively high in the refined RAM. This was due to unexpected hardware and software errors during all stages of the experiment. Future improvement will likely address and solve these issues causing the data exclusion rate to decrease over time. Table 1 summarizes the respective characteristics of the two methods.

**TABLE 1:** Advantages and limitations of the two versions of the radial arm maze.

	Refined radial arm maze	Classic radial arm maze
Overall animal stress level during experiment	Low	Standard
Food restriction	Not required	Required
Required preparation	Transponder implantation	Transponder implantation
Interaction of animal and experimenter	Low to none	High
Daily effort for experimenter	Low (few minutes)	High (hours; depending on number of animals and sessions)
Effort in case of damage/error	High	Low
Effort to analyze data	Low	Low
Measurement of exploratory and day/night activity pattern	Possible	None
Food reward smell masking	Yes	No
Data exclusion	High	Low
Equipment-associated costs	High	Standard

Limitations of our study include the relatively small sample size which, however, was in the range of other studies using the LPS-model to assess long-term cognitive deficits in a radial arm maze. In addition, we used only one disease model. It would be of interest to compare data from additional disease models in the future to further evaluate sensitivity also in terms of group differences. Since the animals in our study were female, it remains to be seen how male mice perform in the refined RAM. Another weakness is the relatively high data exclusion rate. We took a rigorous approach by excluding software output with only small errors. However, minimizing data exclusion remains both a challenge and a goal for future experiments in this setting. This could include prolonging the initial habituation phase in the refined RAM until all animals regularly enter the maze *ad libitum*. Lastly, our study design allowed for up to 10 daily sessions in the refined RAM versus one daily session in the classic RAM. Future studies should carefully consider limiting the number of daily sessions or the time period during which the maze can be entered.

In conclusion, this is the first study to compare a classic manual eight-arm RAM to a fully automated refined setup. While both mazes proved to be solid testing tools, the refined RAM delivered more sensitive and comprehensive data whereas the classic RAM required less data exclusion. The refined RAM therefore represents a valid new method with promising potential in terms of more differentiated data acquisition in a stress-free, voluntary environment for the animal and with only little effort needed by the researcher. In time-sensitive experimental settings which do not allow for flexibility in adjusting the schedule, however, the classic RAM might still be the preferable version. Despite some obvious disadvantages and limitations, the refined RAM constituted a refinement over the classic RAM procedure as it did not require food deprivation or manual handling, thus improving animal welfare. Future studies should demonstrate this in other disease models and further optimize this approach to refine spatial memory tests.

## Data availability statement

The raw data supporting the conclusions of this article will be made available by the authors, without undue reservation.

## Ethics statement

The animal study was reviewed and approved by the State Office for Health and Social Affairs (Landesamt für Gesundheit und Soziales, LaGeSo), Berlin (G290/15).

## Author contributions

JK, JE, ME, SB, and YW designed the study. JK performed the behavioral experiments and analysis. JK, JM, and JE interpreted the data. JK and JE prepared the figures and wrote the manuscript. All authors reviewed the manuscript.

## Footnotes

1
www.randomizer.org
