# Supplementary material for: Assessing spatial learning and memory in mice: Classic radial maze versus a new animal-friendly automated radial maze allowing free access and not requiring food deprivation
Source: Front Behav Neurosci. 2022 Sep 30;16:1013624. doi: 10.3389/fnbeh.2022.1013624 (PMC9562048; doi:10.3389/fnbeh.2022.1013624)
Supplement: Supplementary file 1 [file Data_Sheet_1.PDF]

SUPPLEMENTARY MATERIAL

Supplementary Figure S1

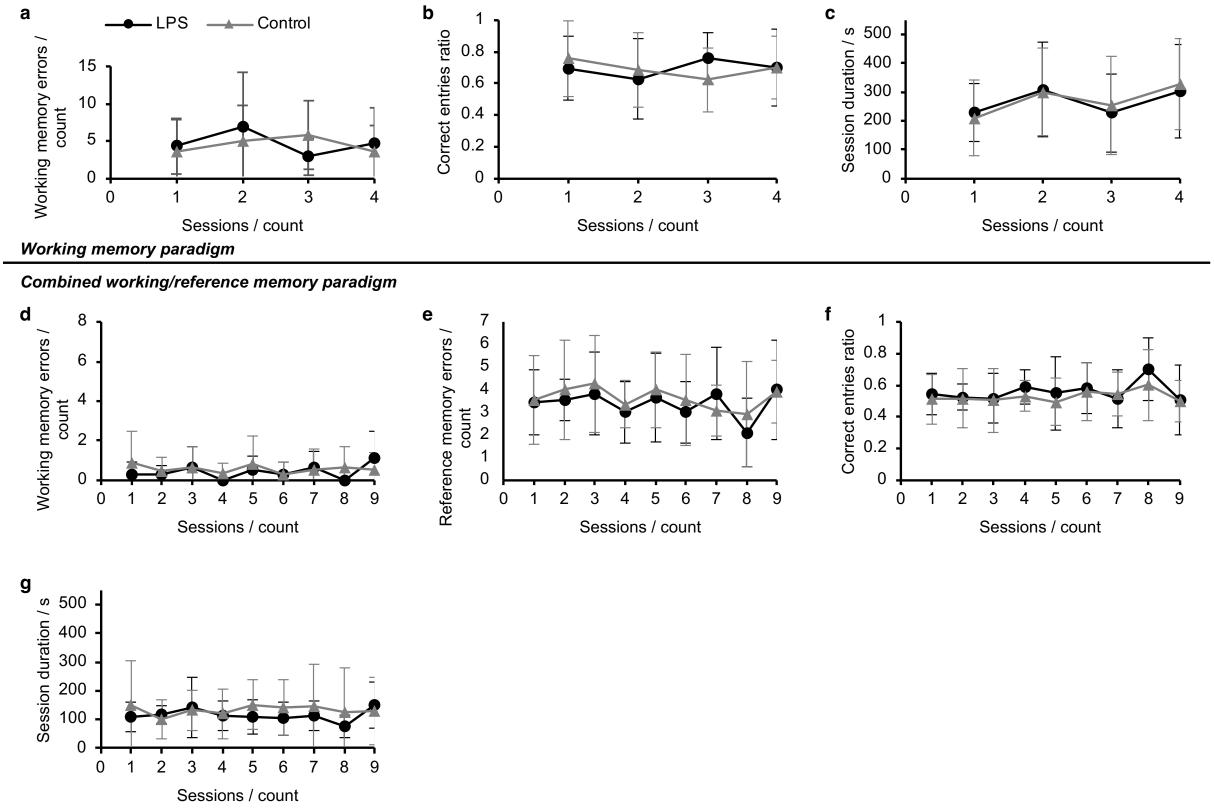

Figure S1. Cognitive performance in the refined RAM per session in the working memory paradigm (a-c) and combined working/reference memory paradigm (d-g). (a) Working memory errors, working memory paradigm; (b) correct entries ratio, working memory paradigm; (c) session duration, working memory paradigm; (d) working memory errors, combined working/reference memory paradigm; (e) reference memory errors, combined working/reference memory paradigm; (f) correct entries ratio, combined working/reference memory paradigm; (g) session duration, combined working/reference memory paradigm. Data are presented as mean (±SD).

Supplementary Figure S2

a

Correct entries ratio (first four arms visited)		Mean	CI (95%)	Difference	CI (95%)	p-value
Day 1						
Refined	LPS	0.47	0.44 - 0.51	0.00	-0.04 - 0.04	0.976
	Control	0.48	0.44 - 0.51			
Classic	LPS	0.45	0.43 - 0.47	-0.02	0.05 - 0.01	0.209
	Control	0.47	0.45 - 0.50			
Day 4						
Refined	LPS	0.48	0.46 - 0.51	0.01	-0.03 - 0.04	0.809
	Control	0.48	0.45 - 0.51			
Classic	LPS	0.47	0.44 - 0.49	-0.02	-0.05 - 0.02	0.311
	Control	0.48	0.46 - 0.51			

0.0 0.2 0.4 0.6 0.8 1.0

b

Correct entries ratio (first eight arms visited)		Mean	CI (95%)	Difference	CI (95%)	p-value
Day 1						
Refined	LPS	0.84	0.77 - 0.92	0.03	-0.07 - 0.12	0.553
	Control	0.82	0.74 - 0.89			
Classic	LPS	0.77	0.71 - 0.82	0.00	-0.08 - 0.08	0.973
	Control	0.77	0.71 - 0.83			
Day 4						
Refined	LPS	0.83	0.76 - 0.89	-0.02	-0.11 - 0.07	0.691
	Control	0.84	0.77 - 0.92			
Classic	LPS	0.82	0.76 - 0.88	-0.05	-0.13 - 0.03	0.231
	Control	0.87	0.81 - 0.93			

0.0 0.2 0.4 0.6 0.8 1.0

Working memory paradigm

Combined working/reference memory paradigm

c

Correct entries ratio (first four arms visited)		Mean	CI (95%)	Difference	CI (95%)	p-value
Day 1						
Refined	LPS	0.53	0.46 - 0.60	-0.02	-0.11 - 0.07	0.604
	Control	0.55	0.48 - 0.62			
Classic	LPS	0.43	0.37 - 0.50	-0.09	-0.18 - 0.00	**0.047**
	Control	0.52	0.45 - 0.59			
Day 4						
Refined	LPS	0.77	0.70 - 0.84	0.08	-0.01 - 0.17	0.078
	Control	0.69	0.62 - 0.76			
Classic	LPS	0.53	0.46 - 0.60	0.01	-0.07 - 0.10	0.754
	Control	0.52	0.45 - 0.59			

0.0 0.2 0.4 0.6 0.8 1.0

d

Correct entries ratio (first eight arms visited)		Mean	CI (95%)	Difference	CI (95%)	p-value
Day 1						
Refined	LPS	0.92	0.88 - 0.97	0.02	-0.04 - 0.08	0.562
	Control	0.90	0.86 - 0.95			
Classic	LPS	0.84	0.79 - 0.88	-0.06	-0.12 - 0.00	**0.040**
	Control	0.90	0.85 - 0.94			
Day 9						
Refined	LPS	0.99	0.95 - 1.04	0.04	-0.02 - 0.10	0.188
	Control	0.95	0.91 - 1.00			
Classic	LPS	0.88	0.83 - 0.92	-0.04	-0.10 - 0.02	0.188
	Control	0.91	0.87 - 0.96			

0 0.2 0.4 0.6 0.8 1

Figure S2. Cognitive performance in the refined and classic radial arm mazes: Correct entries ratio (first four arms visited) and correct entries ratio (first eight arms visited) during the working memory paradigm (a-b) and the combined working/reference memory paradigm (c-d). Separate linear mixed model analyses were conducted. Model-derived estimated marginal means and group differences for (a) the correct entries ratio (first four arms visited) and (b) the correct entries ratio (first eight arms visited) on the first (day 1) and last day (day 4) of the working memory paradigm are shown. Model-derived estimated marginal means and group differences for the combined working/reference memory paradigm on the first (day 1) and last day (day 9) of the paradigm: (c) Correct entries ratio (first four arms visited), (d) Correct entries ratio (first eight arms visited).

Supplementary Figure S3

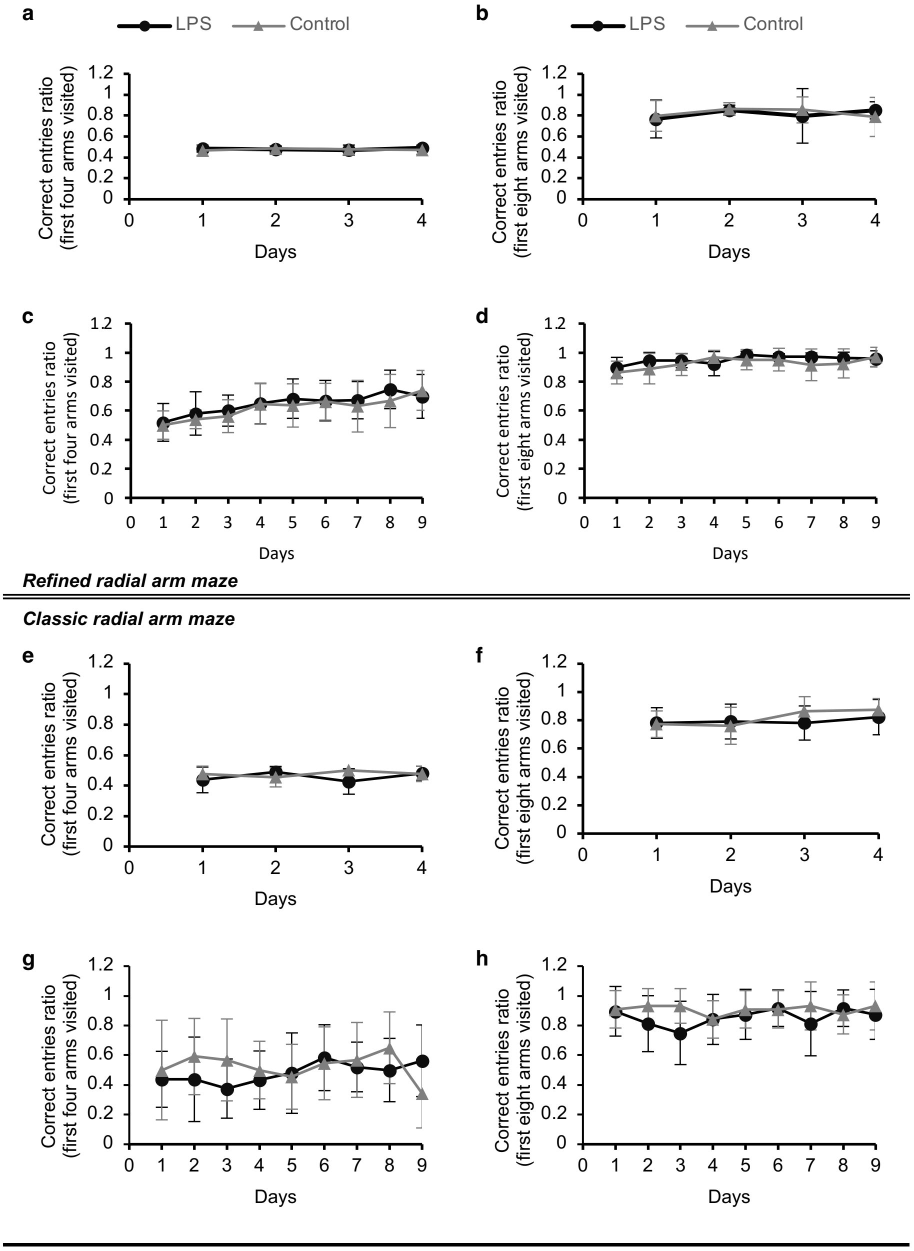

Figure S3. Spatial working and reference memory performance of mice following lipopolysaccharide (LPS)-injection in the refined radial arm maze (RAM) during working memory paradigm (a, b) and combined working/reference memory paradigm (c, d) and in the classic RAM during working memory paradigm (e, f) and combined working/reference memory paradigm (g, h).

Refined RAM: (a) Correct entries ratio (first four arms visited) during the working memory paradigm; (b) correct entries ratio (first eight arms visited) during the working memory paradigm; (c) correct entries ratio (first four arms visited) and (d) correct entries ratio (first eight arms visited) during the combined working/reference memory paradigm. Working memory paradigm: N = 9 (LPS-treated group), N = 6 (control group); combined working/reference memory paradigm: N = 11 (LPS-treated group), N = 11 (control group).

Classic RAM: (e) Correct entries ratio (first four arms visited) and (f) correct entries ratio (first eight arms visited) during the working memory paradigm; (g) correct entries ratio (first four

arms visited) and (h) correct entries ratio (first eight arms visited) during the combined working/reference memory paradigm. N = 12 (LPS-treated group), N = 11 (control group). Data are presented as mean (±SD). Half of the individuals tested with the refined RAM had previously been trained on the classic RAM and vice versa. RAM, radial arm maze.

Supplementary Table S1

Refined RAM

Working memory paradigm

	Difference day 1-day 4	*95 % CI*	*p-value*
**Working memory errors (log)**			
LPS	0.33	-0.2 - 0.86	0.225
Control	0.63	0.06 - 1.19	**0.030**
**Correct entries ratio (all arms visited)**			
LPS	-0.07	-0.19 - 0.06	0.303
Control	-0.12	-0.25 - 0.02	0.081
**Correct entries ratio (first four arms visited)**			
LPS	-0.01	-0.05 – 0.03	0.738
Control	0.00	-0.05 – 0.04	0.880
**Correct entries ratio (first eight arms visited)**			
LPS	0.02	-0.09 – 0.12	0.736
Control	-0.03	-0.14 – 0.08	0.606
**Session duration (log)**			
LPS	0.19	-0.09 - 0.47	0.188
Control	0.44	0.14 - 0.74	**0.004**

Combined working/reference memory paradigm

	Difference day 1-day 9	*95 % CI*	*p-value*
**Working memory errors (log)**			
LPS	0.09	-0.12 - 0.30	0.414
Control	0.19	-0.02 - 0.41	0.073
**Reference memory errors**			
LPS	1.97	1.09 - 2.85	**< 0.001**
Control	1.22	0.34 - 2.10	**0.007**
**Correct entries ratio (all arms visited)**			
LPS	-0.19	-0.27 - -0.11	**< 0.001**
Control	-0.14	-0.22 - -0.07	**< 0.001**
**Correct entries ratio (first four arms visited)**			
LPS	-0.24	-0.34 - -0.14	**< 0.001**
Control	-0.14	-0.24 - -0.04	**0.006**
**Correct entries ratio (first eight arms visited)**			
LPS	-0.07	-0.13 - -0.01	**0.019**
Control	-0.05	-0.11 – 0.01	0.103
**Session duration (log)**			
LPS	0.33	0.13 - 0.53	**0.001**
Control	0.21	0.01 - 0.41	**0.040**

Classic RAM

Working memory paradigm

	Difference day 1-day 4	*95 % CI*	*p-value*
**Working memory errors (log)**			
LPS	0.29	-0.14 - 0.73	0.187
Control	0.59	0.14 - 1.04	**0.010**
**Correct entries ratio (all arms visited)**			
LPS	-0.07	-0.18 - 0.03	0.166
Control	-0.13	-0.23 - -0.02	**0.021**
**Correct entries ratio (first four arms visited)**			
LPS	-0.02	-0.06 – 0.02	0.336
Control	-0.01	-0.05 – 0.02	0.469
**Correct entries ratio (first eight arms visited)**			
LPS	-0.05	-0.14 – 0.03	0.208
Control	-0.10	-0.19 - -0.01	**0.026**
**Session duration (log)**			
LPS	-0.04	-0.27 - 0.19	0.722
Control	0.21	-0.03 - 0.45	0.083

Combined working/reference memory paradigm

	Difference day 1-day 9	*95 % CI*	*p-value*
**Working memory errors (log)**			
LPS	-0.01	-0.21 - 0.20	0.944
Control	0.10	-0.11 - 0.31	0.364
**Reference memory errors**			
LPS	0.89	0.03 - 1.75	**0.042**
Control	0.14	-0.74 - 1.03	0.753
**Correct entries ratio (all arms visited)**			
LPS	-0.06	-0.13 - 0.01	0.102
Control	-0.02	-0.09 - 0.06	0.698

**Correct entries ratio (first four arms visited)**			
LPS	-0.10	-0.19 - 0.00	**0.048**
Control	0.00	-0.1 – 0.10	0.923
**Correct entries ratio (first eight arms visited)**			
LPS	-0.04	-0.1 – 0.02	0.191
Control	-0.02	-0.08 – 0.04	0.579
**Session duration (log)**			
LPS	0.14	-0.06 - 0.33	0.172
Control	0.02	-0.18 - 0.22	0.860

Table S1. Spatial learning in the refined radial arm maze (RAM) and in the classic RAM. Model-based difference between first and last day for LPS-treated and control animals in the working memory paradigm and in the combined working/reference memory paradigm as an assessment of the effect of time on spatial learning. Log, log-transformed data.
